# Tubular Cell Glucose Metabolism Shift During Acute and Chronic Injuries

**DOI:** 10.3389/fmed.2021.742072

**Published:** 2021-10-29

**Authors:** Anna Faivre, Thomas Verissimo, Hannah Auwerx, David Legouis, Sophie de Seigneux

**Affiliations:** ^1^Laboratory of Nephrology, Geneva University Hospitals, Geneva, Switzerland; ^2^Department of Cell Physiology and Metabolism, University of Geneva, Geneva, Switzerland; ^3^Intensive Care Unit, Department of Acute Medicine, Geneva University Hospitals, Geneva, Switzerland

**Keywords:** glycolysis, gluconeogenesis, metabolic shift, acute kidney injury, chronic kidney disease

## Abstract

Acute and chronic kidney disease are responsible for large healthcare costs worldwide. During injury, kidney metabolism undergoes profound modifications in order to adapt to oxygen and nutrient shortage. Several studies highlighted recently the importance of these metabolic adaptations in acute as well as in chronic phases of renal disease, with a potential deleterious effect on fibrosis progression. Until recently, glucose metabolism in the kidney has been poorly studied, even though the kidney has the capacity to use and produce glucose, depending on the segment of the nephron. During physiology, renal proximal tubular cells use the beta-oxidation of fatty acid to generate large amounts of energy, and can also produce glucose through gluconeogenesis. In acute kidney injury, proximal tubular cells metabolism undergo a metabolic shift, shifting away from beta-oxidation of fatty acids and gluconeogenesis toward glycolysis. In chronic kidney disease, the loss of fatty acid oxidation is also well-described, and data about glucose metabolism are emerging. We here review the modifications of proximal tubular cells glucose metabolism during acute and chronic kidney disease and their potential consequences, as well as the potential therapeutic implications.

## Introduction

Renal disease encompasses acute and chronic lesions altering renal physiological function. Chronic kidney disease (CKD) is characterized by an alteration of kidney structure and/or function lasting for more than 3 months (1). Acute kidney injury (AKI) is defined by a sudden increase in serum creatinine or decrease in urine output (2, 3). AKI is recognized as one of the main factors contributing to CKD progression (4, 5) and is associated with significant mortality (6). Although they represent two different diseases by definition, AKI and CKD share many common traits and are very much interrelated (5). Understanding the pathophysiology of both acute and chronic renal injury is mandatory to address the unmet medical need. Tubular cells are important players in AKI and in the progression of CKD. Metabolic modifications and mitochondrial dysfunctions of these cells are key to fibrosis development (7).

The kidney displays the second highest metabolic rate (>400 kcal/kg tissue/day) (8) after the heart. It uses ~7% of the total body daily energy despite its relatively low weight (9). The high energetic needs of the kidney are due on one hand to the transport and active reabsorption of nutrients and electrolytes in the tubules and on the other hand to the active secretion of unneeded compounds (10). Depending on the segment of the nephron, tubular cells use different substrates such as glucose, amino acids, fatty acids, or ketone bodies to produce energy through fatty acid oxidation (FAO) and glycolysis (11, 12). Glycolysis is the process that converts glucose into pyruvate through 10 enzymatic reactions; pyruvate is subsequently converted into acetyl-CoA. FAO is the breakdown of fatty acids at the mitochondrial level that also generates acetyl-CoA. Acetyl-CoA is then used as substrate for the tricarboxylic acid cycle (TCA) and the electron transport chain to produce adenosine triphosphate (ATP). The kidney is also able to perform gluconeogenesis (similarly to the liver), a metabolic pathway that produces glucose from non-hexose precursors. The kidney is therefore both an important consumer of energy and a producer of glucose and participates in systemic glucose metabolism (13).

**Figure 1:**
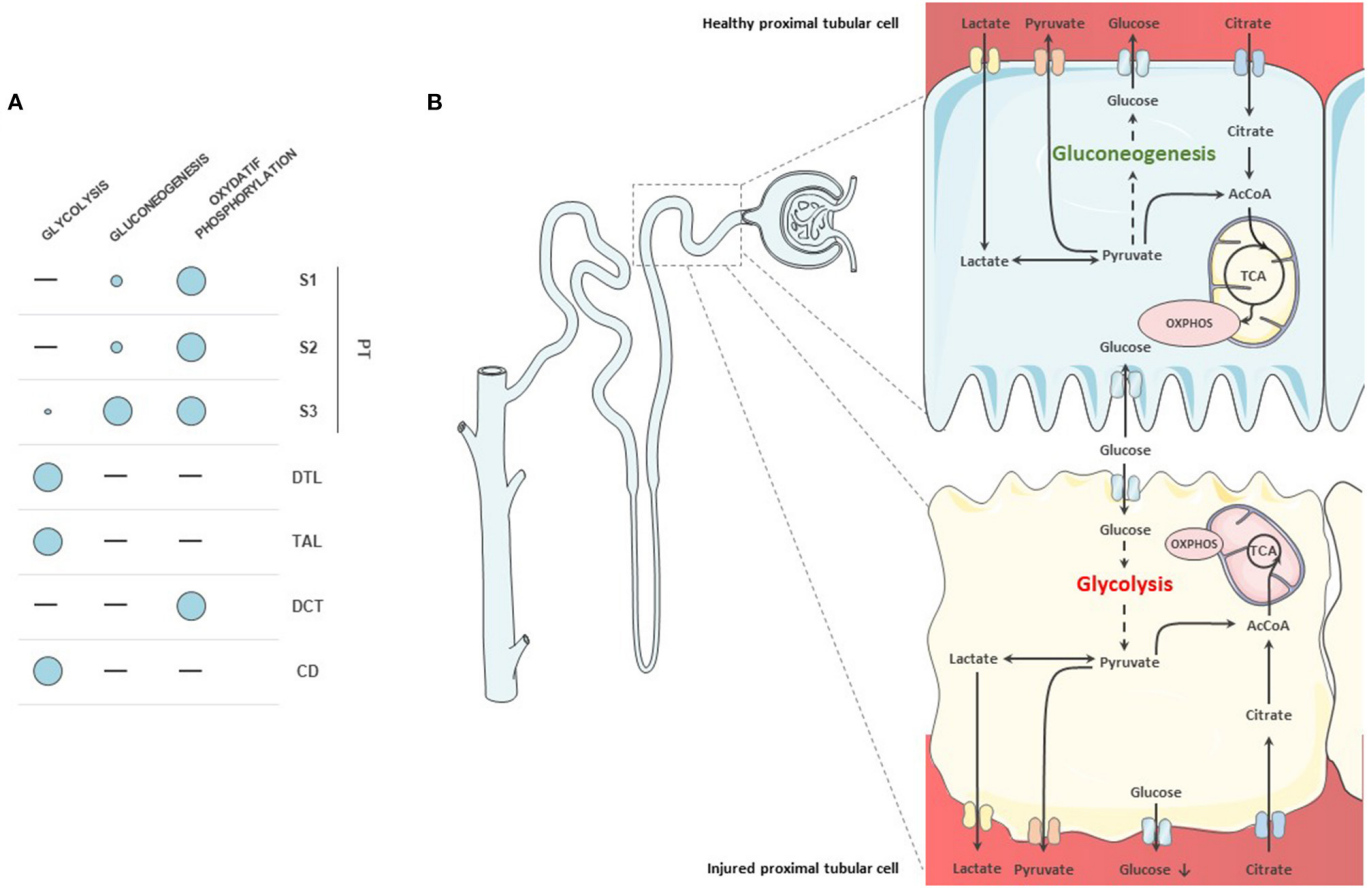
Metabolism of nephron segments. **(A)** Overview of metabolic capacities of tubular cells. Proximal tubular cells do not display glycolytic capacity, except for the S3 segment. They rely on oxidative phosphorylation for their metabolism. They are also able to produce glucose through gluconeogenesis. **(B)** Proximal tubule cells in health and disease. In healthy proximal tubular cells, glycolysis is almost absent although the cells reabsorb glucose. Glucose is produced through gluconeogenesis from pyruvate and released into the circulation. In diseased cells, fatty acid oxidation (FAO) decreases and cells start to use glucose as a substrate. Gluconeogenic abilities are also diminished in injured proximal tubular cells.

Proximal tubular cells (PTCs) are the cells that produce and consume most of the energy in the kidney (14). Under physiological condition, their ability to generate ATP relies mostly on the oxidative phosphorylation of acetyl-CoA from FAO (12). Oxidative phosphorylation allows the production of energy through the transfer of electrons from nicotinamide adenine dinucleotide (NADH) and flavin adenine dinucleotide (FADH) to oxygen, finally leading to ATP production. Besides, PTCs possess the ability to produce energy from a wide range of other substrates, including glutamine, lactate, pyruvate, acetate, citrate, or ketone bodies. PTCs are able to regulate glucose homeostasis by the *de novo* glucose production through gluconeogenesis but also by the reabsorption of large amounts of glucose, mostly *via* SGLT2 on the apical membrane of S1-2 segments and in a lesser extent *via* SGLT1 in the S3 segment. After reabsorption, glucose is released in the blood by glucose transporters (GLUT) (15). However, PTCs are almost unable to use glucose as an energetic substrate, except for the S3 segment (12). This specificity is due to the close localization of the S3 segments to the outer medulla and hence to its strong exposure to changes in O_2_ levels. The complete breakdown of fatty acids is depends on the presence of oxygen and S3 cells therefore have to rely on other energy sources than FAO in case of low oxygen supply (16). On the contrary, the cells in the more distal segments of the nephron display enhanced glycolytic capacity and progressively lose their capacity to perform gluconeogenesis and oxidative phosphorylation. Besides, they can use less varied substrates than the PTCs (10) (Figure 1). Finally, because of their passive blood filtration process glomerular, endothelial and mesangial cells display low rates of oxidative phosphorylation (17). They have a basic glycolytic metabolism, with the ability to perform either aerobic or anaerobic glycolysis, thus producing less energy but with greater efficiency when O_2_ supply is low (18, 19). As a result, they are less sensitive to changes in O_2_ levels than PTCs.

In this review, we will primarily focus on the altered metabolism of PTCs during renal disease. Although several metabolic pathways are interrelated, we here mainly discuss glucose metabolism modifications during kidney disease and its potential local and systemic consequences.

## Glucose Metabolism During Kidney Disease

PTCs are the largest group of tubular cells and are at highest risk of being exposed to hypoxia and other types of injury (20). The importance of these cells in the pathophysiological process of AKI and CKD is now well-established and the specific influence of PTC metabolic alterations in the pathological processes of renal disease has received increasing attention. Among metabolic alterations, mitochondrial dysfunction (21–27) and FAO downregulation (21, 28–30) have been described and already been reviewed (31–36). We here describe in more details the modifications of glucose metabolism in PTCs that occurs during acute and chronic injuries.

**Figure 2:**
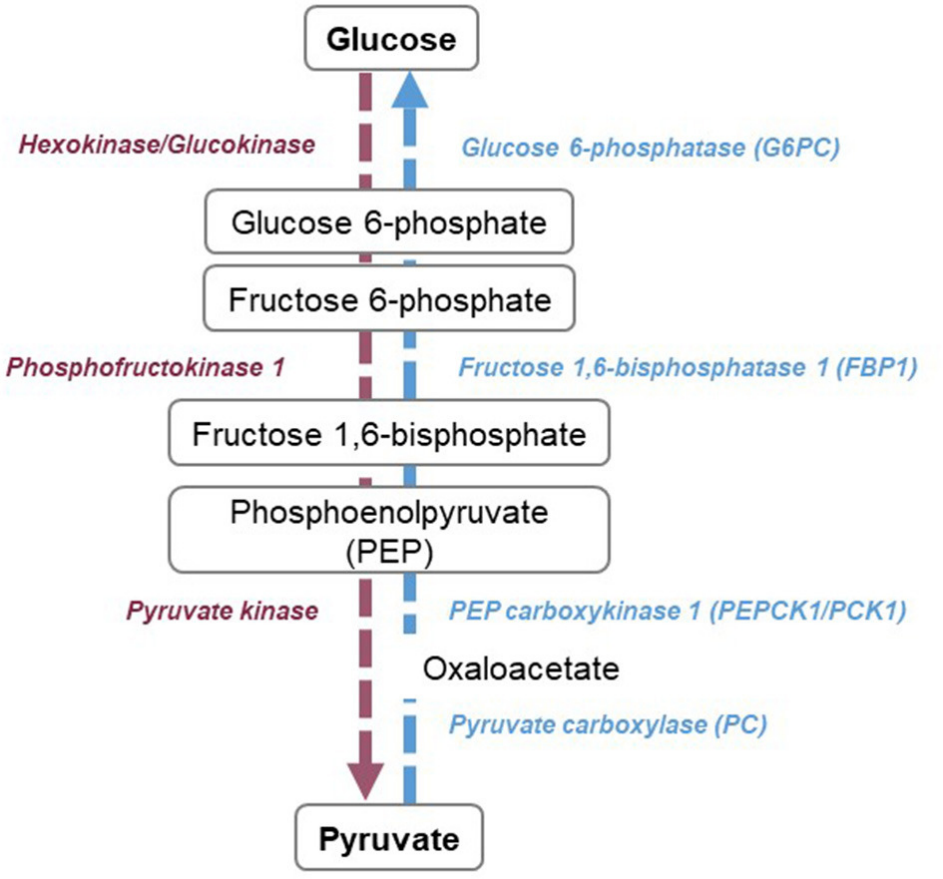
Overview of glycolysis and gluconeogenesis pathways. Glycolysis (purple) consists of 10 reactions, leading to the production of pyruvate. Three steps are considered as rate-limiting, catalyzed by hexokinases or glucokinases, phosphofructokinase 1, and pyruvate kinase. Gluconeogenesis (blue) is the reversed steps of glycolysis, with four specific reactions catalyzed by glucose-6-phosphatase, fructose 1,6-bisphosphatase, phosphoenolpyruvate carboxykinase 1, and pyruvate carboxylase.

Glycolysis is a 10 steps pathway that leads to the production of pyruvate (Figure 2). The first step is catalyzed by hexokinases, which phosphorylates glucose into glucose-6-phosphate (G6P). Glucose phosphate isomerase rearranges G6P into fructose 6-phosphate. Fructose can also be phosphorylated and enter the pathway at this stage (37). Fructose 6-phosphate is then transformed in fructose 1,6-bisphosphate by phosphofructokinase 1, an ATP-dependent reaction and a key regulatory point of the pathway (38, 39). After several steps leading to phosphoenolpyruvate, the pyruvate kinase catalyzes the last reaction, which is also a regulatory point (40). Depending on O_2_ availability, pyruvate is transported into the mitochondria to enter the TCA cycle or in the absence of oxygen, pyruvate is converted into lactate (41). The regulating enzymes of glycolysis are hexokinases, phosphofructokinases, and pyruvate kinases. These three enzymes are activated by the AMP/ADP ratio. Phosphofructokinase is additionally activated by fructose-2,6-bisphosphate and pyruvate kinase by fructose-1,6-bisphosphate. Hexokinase is inhibited by glucose-6-phosphate, phosphofructokinase, ATP, and citrate. Likewise, pyruvate kinase is inhibited by ATP, acetyl-CoA, and alanine (42). Insulin, stimulation of glucose uptake and epinephrine enhance glycolysis (43, 44). Among negative regulators of the pathway are glucagon, cortisol and growth hormone (42, 45, 46).

Physiologically, in conditions of high oxygen supply, fatty acids are mainly taken up by PTCs and used for oxidative phosphorylation to generate ATP. However, AKI is usually associated with a decrease in oxygen supply or relative oxygen deficiency in PTCs. PTCs are hence forced to use glycolysis instead of FAO. This is well-described in different models of AKI, which have shown pyruvate depletion, glycolysis intermediates accumulation and glycolytic enzymes upregulation during this condition (27, 47–51) even at late stages post-reperfusion (27). In chronic disease, analysis of metabolomics and RNA sequencing data from human databases have demonstrated an upregulation of glycolysis, showing a specific metabolic pattern of CKD patients (52, 53). A similar shift toward glycolysis has been observed in obstructive mouse models, with an increased lactate to pyruvate ratio (54).

Gluconeogenesis is a major kidney metabolic pathway, specifically present in healthy PTCs that produces glucose from non-hexose substrates such as lactate, pyruvate, glycerol, or amino acids (55) (Figure 2). In the kidney, lactate is the main substrate of gluconeogenesis and accounts for around 50% of glucose production. It is followed by glutamine (20%) and glycerol (10%) (56, 57). The conversion of pyruvate into glucose is the central pathway of gluconeogenesis. For this, 10 enzymatic reactions are necessary, seven of which are reversible and three that are irreversible. Phosphoenolpyruvate carboxykinase 1 (PCK1) catalyzes the formation of phosphoenolpyruvate from oxaloacetate, fructose-1,6-bisphosphatase 1 (FBP1) catalyzes the hydrolysis of fructose 1,6-biphosphate to fructose 6-phosphate and Glucose 6-phosphatase catalyzes glucose 6-phosphate to D-glucose. Gluconeogenesis plays a crucial role in glycemic homeostasis (58). Indeed, during fasting, once glycogen stores becomes depleted, the body will increasingly rely on endogenous glucose production. Although the liver has for a long time been considered as the sole source of glucose production, the kidneys are responsible for 40% of *de novo* glucose production during the fasted state (13, 59, 60). Renal gluconeogenesis is positively regulated by stress hormones such as hydrocortisone, epinephrine, and norepinephrine (56, 57, 61, 62), which could be linked to a regulation of PCK/Pyruvate Carboxylase (PC) activity (63–65). Insulin on the other hand downregulates renal gluconeogenesis (66), which could be due to PCK1/G6Pc regulation through phosphorylation (66, 67) but also to the reduction of substrates availability (glycerol and glutamine) (68, 69) or their redirection to the oxidative pathways (13, 68). Gluconeogenesis is regulated by factors implicated in global metabolic regulation, such as peroxisome proliferator-activated receptor alpha (PPARα) (70), Forkhead Box O1(FOXO1) (71) and hepatocyte nuclear factor 4 alpha (HNF4α) but also by glucose levels, through the modification of the NAD^+^/NADH ratio which causes an indirect downregulation of PCK1 at mRNA level (66). Acidosis also seems to play a role, with the induction of PCK1 at the mRNA and enzymatic activity level (72, 73) and hence increased renal glucose production.

The regulation of gluconeogenesis during renal pathology was not well-studied until recently. In AKI, a downregulation of gluconeogenesis enzymes was shown in single-cell analysis of mouse AKI as well as in RNA sequencing data from post-transplant AKI patients (74). Alterations of lactate clearance and glucose production were observed using renal catheterism data and rodent experimental models. In a cohort of intensive care unit patients, alterations of metabolism were associated with mortality, underlying the importance of renal glucose metabolism at a systemic level. Overall, gluconeogenesis decreases during AKI and leads to systemic metabolic alterations. In CKD, our preliminary results show similar alterations of gluconeogenesis are observed in a stage specific manner.

Altogether, a metabolic switch from FAO and gluconeogenesis to glycolysis occurs in PTCs during AKI and CKD. To date, the exact cause of this metabolic switch remains unknown. Initially, the switch constitutes a protective mechanism allowing PTCs to maintain energy production in case of low oxygen supply: HIF activation indeed enhances glycolysis through the stimulation of several enzymes of the pathway (hexokinases, glyceraldehyde-3-phosphate dehydrogenase, enolases, phosphofructokinases) and through the activation of glucose transporters (GLUT1-3) (75–77). In later stages of renal disease, inflammation and TGFβ activation could also play an important role in the persistence of the metabolic switch (78, 79); the induction of glycolysis was reproduced by IL-β and c-myc signaling activation (32, 52). In addition, the expression of co-regulators of FAO and glycolysis such as HNF4α or estrogen-related receptor alpha (ESRRA) are modified during AKI (74). Although it is initially cytoprotective, the persistence of the switch may be maladaptive and is associated with a worse kidney prognosis (21, 80, 81). In the second part of this review, we will detail the potential consequences of such metabolic modifications.

## Local Effects of Glucose Metabolism Alterations in the Kidney

**Figure 3:**
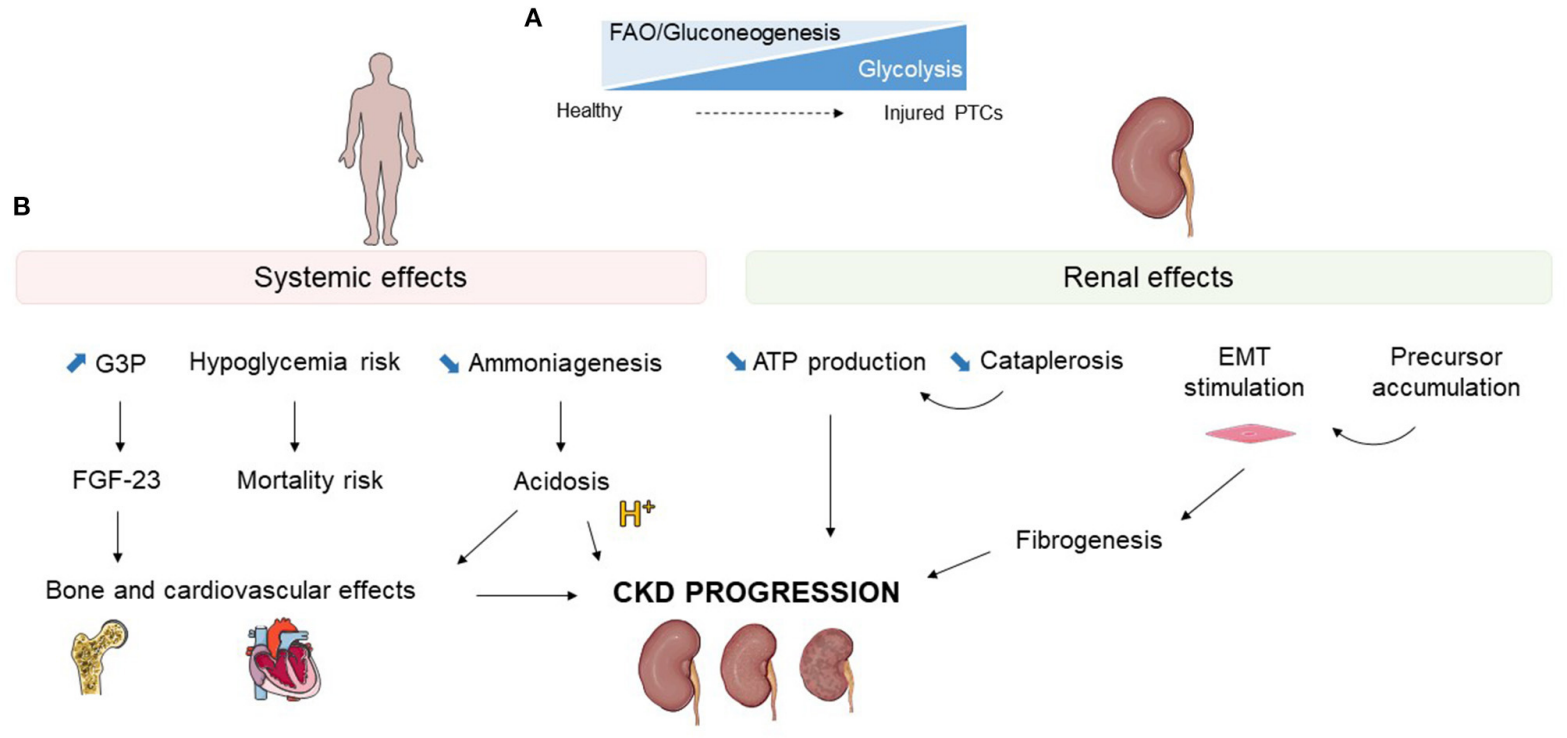
Effects of glucose metabolism alterations during kidney injury. **(A)** Schematic representation of the loss of fatty acid oxidation (FAO) and gluconeogenesis with upregulation of glycolysis occurring in injured proximal tubular cells (PTCs). **(B)** Systemic and renal potential consequences of glucose metabolism modifications during kidney injury. G3P, glycerol-3-phosphate; FGF-23, fibrobastic growth factor 23; EMT, epithelial mesenchymal transition; CKD, chronic kidney disease.

The transition from gluconeogenesis and FAO to glycolysis can have positive or detrimental consequences. Impaired glucose metabolism has been closely linked with mitochondrial dysfunction and a global decrease in energy production (27). The metabolic shift could also directly impact epithelial-mesenchymal transition (EMT) and fibrogenesis and therefore enhance CKD progression. Finally, the accumulation of metabolic precursors may influence renal disease (Figure 3).

### Energy Production

As described earlier, when oxygen supply decreases, PTCs start using mostly anaerobic glycolysis to meet their energy demand. This change results in a decrease in ATP production and thus a diminished energy availability. Indeed, compared with the complete metabolism of fatty acids, which generates 106 ATP, aerobic glycolysis produces 36 or 38ATP and anaerobic only only 2 ATP (14, 82). Enhanced glycolysis may in addition have a detrimental effect on mitochondrial function (83). Phosphokinase mutase 2 (PKM2), one of the glycolytic enzymes, may for example promote mitochondrial fusion (84). Inhibition of gluconeogenesis also influences the TCA cycle. Indeed, gluconeogenesis plays a role in cataplerosis, a crucial pathway for mitochondrial function. Cataplerosis consists of the removal of TCA cycle intermediates and is necessary to maintain the cycle's function (85). The gluconeogenic enzyme PCK1/2 catalyzes the reaction of oxaloacetate to phosphoenolpyruvate, a major reaction of the cataplerotic pathway (86). Downregulation of gluconeogenesis enzymes could for this reason lead to impaired cataplerosis and hence impact the TCA cycle (86), as has been shown in other organs (87).

Thus, although enhancing glycolysis and decreasing gluconeogenesis may initially spare oxygen and maintain ATP production, on the longer term these regulations may lead to further blockade of the TCA cycle and mitochondrial dysfunction, which will affect global energy production and therefore PTC function.

### Fibrogenesis and CKD Progression

Modifications of tubular metabolism is associated with CKD progression and fibrosis. For instance, the decreased ability to perform FAO is associated with kidney fibrosis progression and reversing this loss by different approaches appears nephroprotective (21, 24, 29, 88). Regarding glucose metabolic enzymes, PKM, hexokinases, phosphofructokinase 1, and enolase are able to promote EMT *via* different mechanisms, as reviewed elsewhere (89). In cancer research, FBP1 expression, a key gluconeogenetic enzyme, was inversely linked with Snail activation, a major activator of EMT, whereas FBP1 overexpression protected from EMT (90).

#### In Acute Kidney Injury

Enhancing glycolytic capacity induces a reprogramming of the somatic cells that promotes tubular regeneration (91, 92) and spares more valuable substrates such as fatty acids or amino acids which could then be used for cell regeneration (93). Increased glycolysis also generates more NADPH and glutathione, thus decreasing oxidative stress (48, 94). Similarly, decreasing gluconeogenesis may spare kidney energy in conditions of stress, hence promoting cell survival. Altogether, glycolysis improves PTC survival initially. Nevertheless, the persistence of glycolysis after the acute phase could be detrimental for kidney function. Given this dual impact of glycolysis on PTCs, interventional studies with glycolysis modulation yielded mitigated results. In PTCs, an activation of glycolysis through the inhibition of TP53-inducible glycolysis and apoptosis regulator (TIGAR) is protective in ischemic AKI (93). On the contrary, decreasing glycolysis through the knockout of Nod-like receptor (NLR) family member X1 (NLRX1) was shown to be detrimental (95). Inhibition of PKM through the S-nitroso-CoA Reductase system or fructokinase blockade were also protective during AKI (96, 97). SGLT2 inhibitors have shown the ability to suppress aberrant glycolysis in proximal tubules (98) and stimulate gluconeogenesis (99). Even though some associations between SLGT2 inhibitors and AKI were reported in the US Food and Drug Administration Adverse Event Report System (100), subsequent meta-analysis of large clinical studies indicate a favorable safety profile (101) or even a reduction of the AKI risk (102–104). In preclinical models, some data indicate a protective role (105, 106). Finally, despite many confounding factors, stabilization of HIF, a key glycolysis promoter, is protective in AKI (91, 92, 107).

#### In Chronic Kidney Disease

In diabetic kidney disease, the switch to anaerobic glycolysis is well-described (108) and associated with CKD progression (109). In other CKD models, a reduction of glycolysis appears in contrast to be rather protective overall. PKM activation was shown to be favorable in a diabetic kidney disease model (109) but induced interstitial fibrosis in other models (110). Fructokinase blockade was also protective in diabetic nephropathy (111, 112). Glycolysis inhibitors (shikonin and 2-deoxyglucose) demonstrated attenuated fibrosis in an obstructive CKD model (113). In the same model, deletion of Tuberous sclerosis complex 1, a key regulator of glycolysis, induced glycolysis and enhanced fibrosis (114). In diabetic mice models, the suppression of SIRT3, a major mitochondrial enzyme involved in central metabolism activating many oxidative pathway, was stimulated the fibrogenic kidney pathway (80). Glycolysis inhibition with 2-deoxyglucose alleviated the phenotype (80, 115). As described before, SGLT2 inhibitors seem to decrease glycolysis and enhance gluconeogenesis (98, 99); their major protective effect in CKD is well-established and could partially rely on the modulation of the metabolic switch. In the polycystic model of CKD, characterized by marked cellular proliferation, numerous studies observed that glycolysis blockade is nephroprotecive (115–117).

In sum, the timing of glycolysis induction may be the reason of these contradictory results (27). Glycolysis enhancement and gluconeogenesis loss in PTCs during AKI may promote cell survival and proliferation in an initial phase but their persistence could favor fibrosis progression in the kidney in a more chronic phase. The type of kidney injury could also explain the difference in experimental results.

### Precursors Accumulation

Metabolic pathway blockade leads to the accumulation of precursors, which may overload the cells and contribute to inflammation and fibrosis. Among these precursors, some also have direct toxic effects; blocking FAO will for example increase intracellular concentrations of fatty acids. This results in a toxic environment, impacting ATP production and inducing apoptosis (14). Flux analysis of metabolites indicate that the kidney also plays a major role in clearing circulating citrate and lactate (118, 119); in the case of gluconeogenesis blockade, as observed in AKI, a decrease in lactate clearance occurs (74). Although some biases exist, lactate clearance has been associated with mortality in AKI (120), as well as metabolic alterations (74). Recent papers indicate a potential toxic role of tubule-derived lactate, with increased fibrogenesis (121). Indeed, the inhibition of lactate production *via* glycolysis inhibition was able to reduce the activation of fibroblasts in a model of folic acid-induced AKI. In CKD, the effects of gluconeogenesis decrease are less described but can be inferred from enzymatic deficits. Deficiency in glucose-6-phosphatase (G6PC), a major gluconeogenesis enzyme, has been extensively studied in humans with glycogen storage disease type I. These patients are especially prone to CKD development, due to lipid and glycogen accumulation in the kidneys and the activation of the renin-angiotensin system. G6PC knock-out mice also develop renal cysts. G6PC deficiency seems to be linked with the loss of polycystic kidney genes and HNF1B (122).

## Systemic Effects of Glucose Metabolism Alterations in the Kidney

Besides its potential effects on the outcome of kidney disease, modifying renal glucose metabolism has a systemic impact. Alteration of tubular cell metabolism may also imply release of new metabolites that can affect unexpected systemic functions (Figure 3).

### Hypoglycemia Risk

In a fasting state, once glycogen stores are depleted, gluconeogenesis becomes increasingly important and represents up to 90% of total glucose production after 40 h of fasting in order to maintain normoglycemia (123). In fasting conditions, the kidney is able to provide up to 40% of systemic glucose (13, 59, 60).

As demonstrated recently, the enhancement in glycolysis and decrease in gluconeogenesis in AKI leads to a negative renal glucose balance and increases hypoglycemia risk (74). The role of glucose production of the kidney has probably as much importance in CKD as in AKI. In the ACCORD study, diabetic patients with low renal function displayed a higher risk of hypoglycemia and mortality (124). Similarly in another study, both diabetic and non-diabetic patients with CKD had an increased risk of in-hospital hypoglycemia (125). Although not clearly described or studied in CKD, the enhanced risk could be partly related to loss of gluconeogenesis.

### Lactate and Acid-Base Homeostasis

In the kidney, gluconeogenesis uses lactate as its main substrate. In renal arteriovenous catheterization experiments, impaired renal gluconeogenesis due to AKI induced a decrease in glucose production and lactate clearance. The increased lactate and lower glucose levels were associated with higher mortality in a retrospective cohort of critically ill patients (74).

Changes in glucose metabolism impacts acid-base regulation as illustrated by the close correlation between gluconeogenesis and ammoniagenesis. To counterbalance acidosis, the kidney generates ammonia, mainly from glutamine deamination, which forms α-ketoglutarate (α-KG) and NH4^+^ *via* the ammoniagenesis pathway (126, 127). After its uptake by PTCs, glutamine is catalyzed into glutamate. Glutamate is then converted to α-ketoglutarate, the carbon skeleton of glutamine. α-KG is further metabolized and is ultimately transformed into glucose, through the gluconeogenic pathway (128). Renal ammoniagenesis and gluconeogenesis are hence two closely interdependent pathways. Despite the ability of all segments to produce ammonia, metabolic acidosis will only enhance ammoniagenesis in the proximal segments S1 and S2 (127), a process which is altered in AKI and CKD. As the complete metabolism of glutamine requires PCK1 activity (129), gluconeogenesis alterations may participate in the loss of ammoniagenesis abilities of PTCs and hence decrease their defense against metabolic acidosis.

### FGF23 and Glycerol-3-Phosphate

Local changes of metabolism lead to modification of arterial and venous kidney metabolite profile. Fibroblast growth factor 23 (FGF23) is a protein implicated in vitamin D metabolism and phosphatemia regulation. FGF23 levels increase with any type of kidney injury; in AKI, in early and late CKD, and could be linked with cardiovascular mortality (130–132). Despite that, the regulatory factors of FGF23 during AKI and CKD are poorly described. A recent study identified glycerol-3-phosphate (G3P) renal venous production as the most predictive factor for FGF23 elevation in AKI, which was confirmed experimentally (133). In human and rodent with AKI, G3P levels increased rapidly, a process mediated by glycerol-3-phosphate acyltransferase and lysophosphatidic acid (LPA) (133). G3P can arise from three mechanisms, one being the glycolytic pathway *via* the conversion of dihydroxyacetone phosphate in G3P glucose (134). As glycolysis increases during AKI, it may explain these results and outlines the importance of renal glucose metabolism during AKI and its potential systemic effects. A second pathway for G3P production is from glycerol, which is indirectly linked to gluconeogenesis as PCK1 is involved in glycerol production. As glycolysis and gluconeogenesis are also dysregulated during CKD, similar effects could be expected in this condition. Therefore, kidney metabolic switches may impact pathways as diverse as mineral metabolism and heart hypertrophy, both processes being regulated by FGF23 (135). This example shows how local metabolic change in PTCs can influence very diverse systemic pathways, many of which are probably still unknown to this date.

## Conclusion and Perspectives

Metabolic adaptation of renal cells during acute or sustained injury is a complex mechanism. The metabolic switch from fatty acid oxidation and gluconeogenesis toward glycolysis is part of a cytoprotective stress mechanism. However, persistence of this metabolic profile is associated with fibrogenesis. Glucose metabolism impairment could have a broad impact, ranging from local to systemic consequences. Interestingly, renal glucose metabolism is amenable to therapeutic interventions.

## Author Contributions

AF reviewed the literature, drafted the manuscript, and drew the figures. TV reviewed the literature, edited the manuscript, and drew the figures. HA reviewed the literature and edited the manuscript. DL edited the manuscript and the figures. SS designed and supervised the project, edited the manuscript and the figures. All authors contributed to the article and approved the submitted version.

## Funding

AF was the recipient of a grant from the Swiss National Science Foundation (SNSF 323530_191224) and from the Carlos et Elsie de Reuter Founding. SS was supported by grants from the Swiss National Science Foundation (SNSF PP00P3-187186/1), Jules Thorn Foundation and NCCR Kidney.ch.

## Conflict of Interest

The authors declare that the research was conducted in the absence of any commercial or financial relationships that could be construed as a potential conflict of interest.

## Publisher's Note

All claims expressed in this article are solely those of the authors and do not necessarily represent those of their affiliated organizations, or those of the publisher, the editors and the reviewers. Any product that may be evaluated in this article, or claim that may be made by its manufacturer, is not guaranteed or endorsed by the publisher.
